# ^1^H NMR and EPR Spectroscopies Investigation of Alginate Cross-Linking by Divalent Ions

**DOI:** 10.3390/ma16072832

**Published:** 2023-04-02

**Authors:** Anna A. Forysenkova, Valeria A. Ivanova, Inna V. Fadeeva, Georgy V. Mamin, Julietta V. Rau

**Affiliations:** 1 A.A. Baikov Institute of Metallurgy and Material Science, Russian Academy of Sciences, Leninsky Avenue 49, 119334 Moscow, Russia; 2 Phystech-School of Electronics, Photonics and Molecular Physics, Moscow Institute of Physics and Technology, Institutsky Lane 9, 141701 Dolgoprudny, Russia; 3 Institute of Physics, Kazan Federal University, Kremlevskaya 18, 420008 Kazan, Russia; 4 Istituto di Struttura della Materia, Consiglio Nazionale delle Ricerche (ISM-CNR), Via del Fosso del Cavaliere 100, 00133 Rome, Italy

**Keywords:** alginate, alginate cross-linking, alkaline earth metals, transition metals, nuclear magnetic resonance, electron paramagnetic resonance

## Abstract

Alginate is a natural polymer widely applied in materials science, medicine, and biotechnology. Its ability to bind metal ions in order to form insoluble gels has been comprehensively used to create capsules for cell technology, drug delivery, biomedical materials, etc. To modify and predict the properties of cross-linked alginate, knowledge about the mechanism of alginate binding with metal ions and the properties of its gels is necessary. This article presents the results obtained by proton Nuclear Magnetic Resonance Spectroscopy for alginate containing calcium and strontium (alkaline earth metal diamagnetic) ions and by Electron Paramagnetic Resonance Spectroscopy for alginate with copper (Cu) and manganese (Mn) (transition metal paramagnetic) ions. It was found that in the case of calcium (Ca) and Mn ions, their concentration does not affect their distribution in the alginate structure and the cross-linking density. In the case of strontium (Sr) and Cu ions, their number affects the number of binding sites and, accordingly, the cross-linking density. Thus, the cross-linking of alginate depends mainly on the characteristics of specific cations, while the nature of the bond (ionic or coordination type) is less important.

## 1. Introduction

Alginate is a linear anionic polysaccharide obtained from brown algae or bacteria and consisting of repeating units of β-1,4-linked D-manuronic acid (M) residue and L-guluronic acid (G) residue in various ratios [1]. Alginate molecules either contain acid residues GMGM…GMGM or fragments of repeating acid residues in blocks GG…GMM…MG … [2]. It is the order and ratio of these blocks that determine the properties of a particular alginate [1,2].

Alginate GG-blocks interact with divalent cations that bind to two opposite GG-blocks in an orderly manner, forming “egg-box” conformations, which leads to the formation of an insoluble network [1]. The higher the content of GG-blocks, the more opportunities for cross-linking of alginate by this mechanism and the formation of rigid hydrogels occur [2]. With a high content of MM-blocks, alginate forms less rigid structures with different mechanical, rheological, and other properties [2].

For biotechnology and pharmaceutical use, alginate hydrogels should possess properties suitable for cell capsules and containers for drug delivery technology [3]. In addition, alginate can be applied to the development of biocompatible and biodegradable materials for wound dressing, anti-adhesion barriers, and orthopedic implants [2,4,5]. In these areas of application, the goal is to obtain alginate materials with appropriate characteristics of mechanical strength and bioresorption [5,6]. 

Alginate is known to decompose under the action of the enzyme alginate lyase, which can be found in algae, marine invertebrates, and microorganisms [7,8]. At the same time, ionically cross-linked alginate is not subjected to enzymatic dissolution [8]. In the human body, the bioresorption of cross-linked alginate strongly depends on the presence and activity of anions, such as lactates, citrates, and phosphates, competing for calcium cations [8]. These processes cannot be controlled from the outside, but it is possible to control the degree of alginate cross-linking, thereby setting certain mechanical properties of materials and predicting the rate of bioresorption [9]. 

A number of studies have been devoted to the investigation of the mechanical [10,11,12], physicochemical [13], and biological properties of cross-linked alginate gels [1,14,15]. The processes of alginate cross-linking with alkaline earth metal ions and transition metal ions were studied using ^1^H and ^13^C NMR spectroscopy in solutions and solids [15,16,17,18,19,20,21,22,23]. In addition, by means of the Density Functional Theory (DFT) of 3D models, the data on the features of the mechanisms and binding energies of various metals with oxygen in the alginate structure were obtained [24,25], and general conclusions about the interaction of alginate with polyvalent ions were made. The objective of the present work is to study and compare the cross-linking of alginate with a number of alkaline earths and transition metals by means of proton Nuclear Magnetic Resonance Spectroscopy (^1^H NMR) and Electron Paramagnetic Resonance Spectroscopy (EPR), and compare the data obtained by both methods. It should be noted that to the best of our knowledge, in this work, for the first time, the EPR spectroscopy was applied to investigate the cross-linking of alginate by metal ions. The obtained results can be useful not only for the development of new biocompatible materials for medicine, but also for biosensor applications [16,26]. 

## 2. Materials and Methods

### 2.1. Preparation of Alginate Samples

To register the ^1^H NMR spectra of alginate, stock solutions of alginate (ALG) (pure, Reakhim, Moscow, Russia) 1 mL of 1% wt. alginate in D_2_O (GmbH, Kastellaun, Germany) was taken and further diluted. An aliquot of 100 μL was taken with a pipette and diluted to 500 μL with the addition of D_2_O to obtain 0.2% wt. alginate solution. Separately, solutions of cross-linking cations were prepared in D_2_O: CaCl_2_ (chemical grade, Chimmed, Moscow, Russia), Sr(NO_3_)_2_ (pure, Lenreactiv, Saint Petersburg, Russia), Zn(NO_3_)_2_·4H_2_O (chemical grade, Chimmed, Moscow, Russia), CoCl_2_·6H_2_O (pure, Lenreactiv, Saint Petersburg, Russia). A number of metals were selected based on the bond strength with alginate [17,25]. 

To a solution of 0.2% wt. alginate, a solution of a cross-linking cation in various amounts was added (Table 1). The amount of cross-linking ion influences the viscosity of alginate gels, and, consequently, the possibility of the ^1^H NMR spectra recording [19].

To study the cross-linking of alginate by EPR spectroscopy, solid samples were obtained in the form of films formed from 2.5% wt. alginate solution. A number of 150 ± 10 µm thick strips were used in the experiments. The presence of paramagnetic impurities in the samples was not detected. The samples weighing from 2.5 to 80 mg were immersed in a 400 μL 102.5 mmol/L solution of Cu(NO_3_)_2_·4H_2_O (analytical grade, Chimmed, Russia) or Mn(CH_3_COO)_2_·4H_2_O (analytical grade, Chimmed, Moscow, Russia) and kept in a BioSan MSV-3500 vortex at 1200 rpm for 1 h. Afterward, the samples were washed in distilled water and dried for 24 h.

Reagents for ^1^H NMR and samples for EPR were weighed using a Vibra HT balance (accuracy of ±0.5 mg), and the solution volume was measured with a Sartorius 1000 μL micropipette. Mixing of the solution with the alginate sample was carried out in a BioSan MSV-3500 vortex at 1200 rpm.

### 2.2. Liquid State ^1^H MNR Spectroscopy Study

All ^1^H NMR spectra were recorded at 25 °C on a Varian Inova 500 NMR spectrometer (operating frequency for protons of 500 MHz) equipped with a 5 mm AutoX DB probe, included in the standard complete set. The MestReNova 14.2.1 software was used to process the ^1^H NMR spectra. The spectra were obtained by averaging 128 independent scans. The spectral width was 8000 Hz (16 ppm), the size of the real spectrum—65.5 K data points, the acquisition time—5 s, and the pulse length—11 µs.

### 2.3. EPR Spectroscopy Study

The X-band (9.64 GHz) EPR spectra were registered with Bruker ElexSys 500/580 EPR spectrometer at 25 °C in continuous wave mode with a magnetic field modulation amplitude of 0.2 mT and modulation frequency of 100 kHz. The number of paramagnetic centers was measured following a standard method described in [27], by comparing the integrated intensity of the EPR lines of the measured sample and the integrated intensity of the Cu(DETC)_2_ solution spectrum with Cu ions number of 7.2 × 10^−9^ mol. The Cu complex with diethyldithiocarbamate (DETC)—Cu(DETC)_2_ solution—is a standard reagent used in the EPR spectroscopy for comparison and calibration.

## 3. Results and Discussion

### 3.1. Liquid State ^1^H MNR Spectroscopy Results

To assess the nature of cross-linking by the ^1^H NMR spectroscopy, we chose divalent cations of alkaline earth metals, such as calcium (Ca) and strontium (Sr), and cations of transition metals, such as cobalt (Co) and zinc (Zn). Alginates, when dissolved in water, form gels, which become more viscous with the increase in the number of cross-linking ions. This reduces the resolution of the ^1^H NMR spectrum. Therefore, we chose sufficiently low concentrations of the initial sodium alginate and cross-linking ions, which made it possible to obtain resolved signals, in agreement with the results published in [19]. Because of this, the amount of substance in the test solution was fairly little, and the presence of H_2_O in the alginate resulted in a high-intensity water proton signal in the spectrum. As a result, several of the alginate proton signals up to 4.3 ppm were suppressed by the high-intensity water signal. 

**Figure 1:**
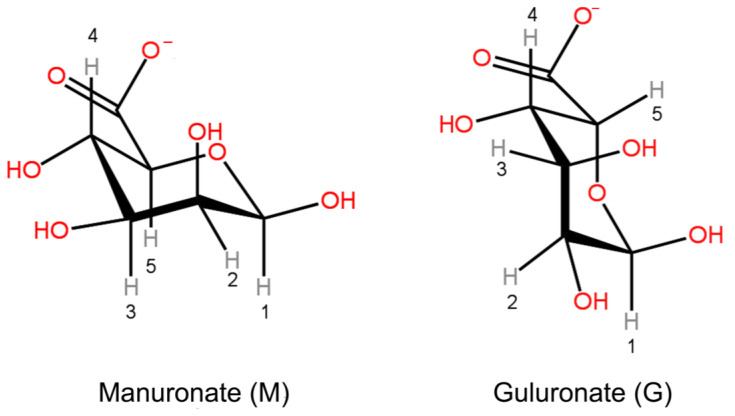
Manuronate and guluronate residues structural formulas.

**Figure 2:**
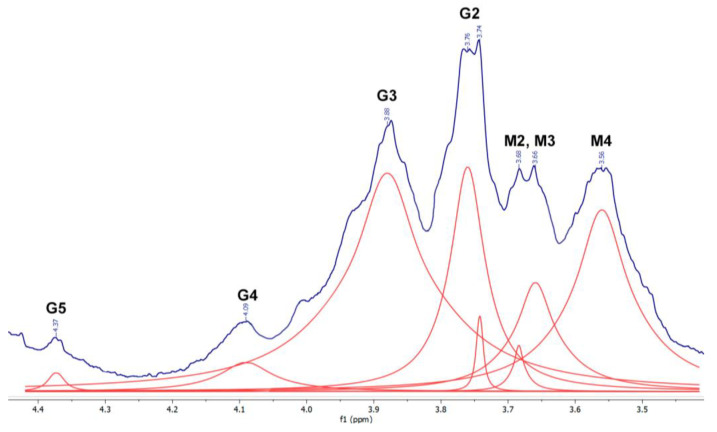
^1^H NMR spectrum of ALG. Fitting is represented by red lines, fitting baseline is shown at the bottom.

For the sake of clarity, the structures of manuronate and guluronate residues with protons designation (only for protons observed in the ^1^H NMR spectra) are presented in Figure 1. The obtained spectrum of ALG, including signals from protons G2, G3, G4, and G5, i.e., protons closest to the carboxyl group of guluronate residue, as well as protons M2, M3, and M4 of manuronate residue, is shown in Figure 2. 

**Figure 3:**
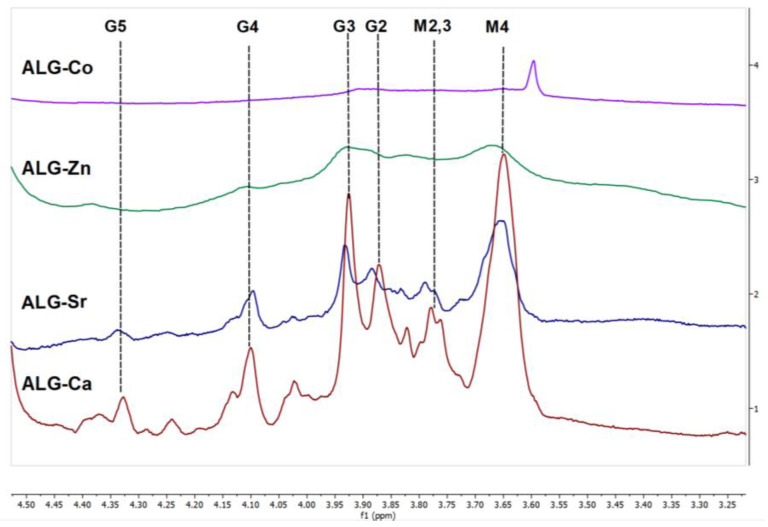
^1^H NMR spectra of alginate cross-linking by Ca, Sr, Zn, and Co ions.

Figure 3 demonstrates the spectra of ALG cross-linking by the Ca, Sr, Zn, and Co ions. In the case of ALG-Zn (0.1 µmol), the broadening and merging of signals can be observed. Apparently, this resulted from an increase in viscosity and formation of a viscous gel due to the strong coordination covalent bonds of Zn with oxygen atoms in both the M and G residues, in agreement with [23]. The impossibility of obtaining the spectrum for ALG-Co (Figure 3) is most likely associated with the paramagnetic characteristics of Co^2+^, leading to signal broadening and merging. 

Taking into account the obtained data, Ca^2+^ and Sr^2+^ cations were chosen for further research on the alginate cross-linking.

**Figure 4:**
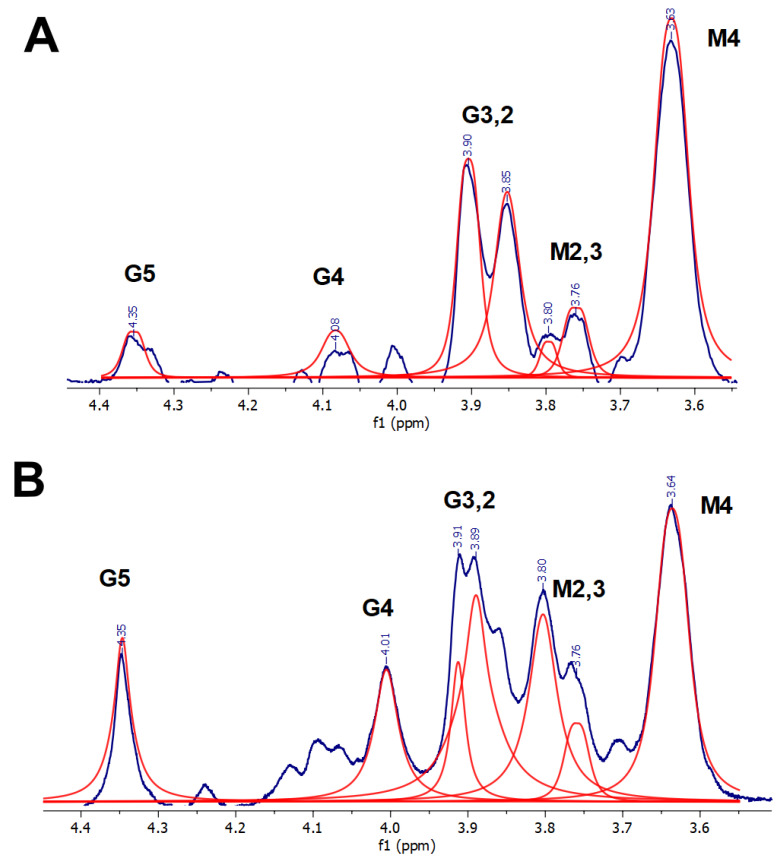
^1^H NMR spectra of (**A**) ALG–Sr and (**B**) ALG–Ca, cross-linked by equal quantities (0.3 μmol) of Ca^2+^ and Sr^2+^ ions, respectively. Fitting is represented by red lines, fitting baseline is shown at the bottom.

Figure 4 shows the spectra of alginate cross-linked with the same quantities of Ca and Sr (Figure 4A,B, respectively). To compare the spectra of ALG-Ca and ALG-Sr, we normalized the intensities of all the signals in each spectrum to a well-resolved and stable M4 signal (H4 protons of the M residue), in agreement with the previously reported results in [22]. Its shape and chemical shift did not vary during all the series of spectra, indicating the signal’s stability. The signals of G and M residue protons differ. The ALG-Sr spectrum (Figure 4A) demonstrated the resolution of the G2 and G3 signals, but with a lower relative intensity, compared to the ALG-Ca spectrum (Figure 4B), while the signals M2, M3, G4, and G5 had a low intensity. The obtained spectra may indicate the predominance of the Sr bond with the GG and GM residues. Although a scheme was proposed, in which Sr binds exclusively to the GG residue [3], forming an “egg-box” structure, according to our data, it is likely that the coordination of the Sr ion to the GM sites takes place as well resulting in the formation of a “pocket-like” structure, shown for Mn in [21]. It was noted that such a configuration is characteristic of divalent cations [21]. Thus, upon alginate cross-linking with the Sr ions, the latter are coordinated with a greater number of oxygen sites than the Ca ions.

In the spectrum of ALG-Ca (Figure 4B), the intensity of both the G and M2,3 signals is comparable to the intensity of the M4 normalization signal. This may indicate that, at a given number of ions, the G and M residues are equally involved in Ca binding. In this case, weak ionic bonds between COO^-^ and Ca or Sr ions are formed [27].

**Figure 5:**
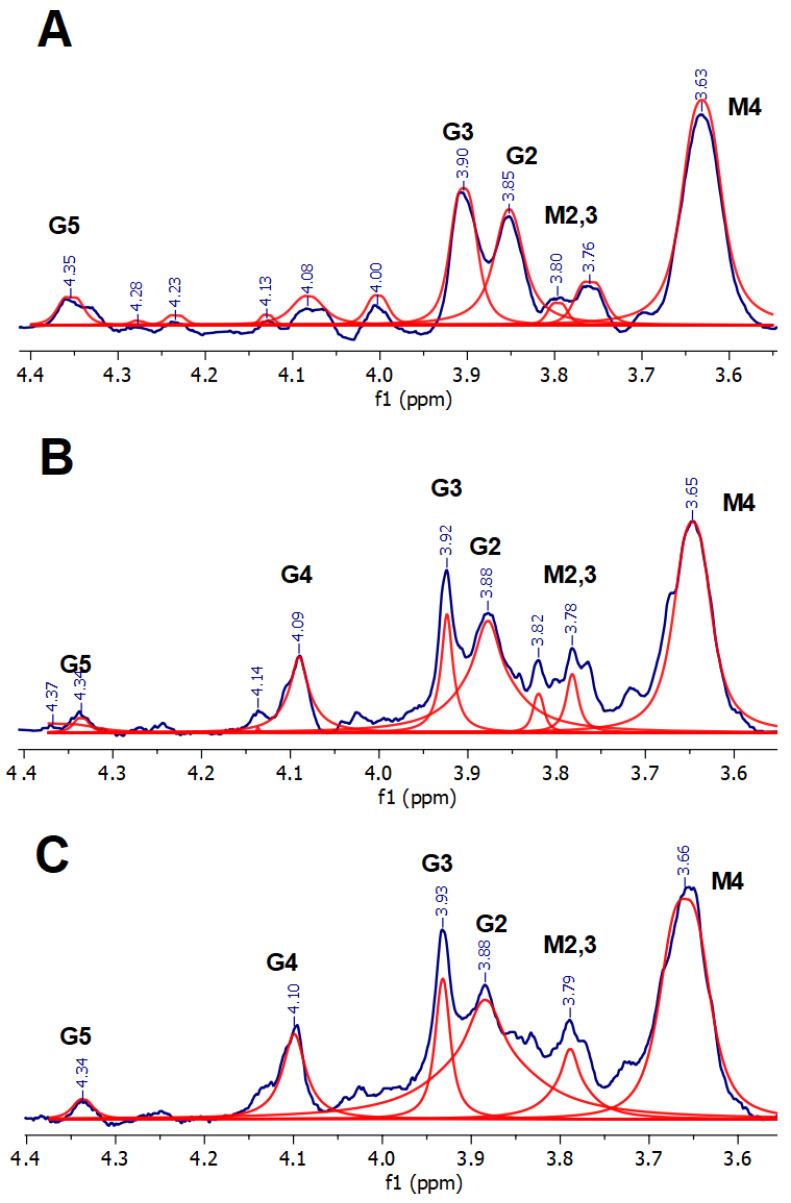
^1^H NMR spectra of ALG-Sr, cross-linked by different quantities (μmol) of Sr^2+^: (**A**) 0.3 μmol, (**B**) 1 μmol; (**C**) 2.5 μmol. Fitting is represented by red lines, fitting baseline is shown at the bottom.

As the quantity of the Sr^2+^ ions increased, the spectrum of ALG-Sr changed (Figure 5). At 1 µmol of Sr, the G4 proton signal became more intense. This may be due to the redistribution of Sr^2+^ ions in the alginate structure with an increase in their number. At a low quantity of Sr^2+^ ions, they form “egg-box” structures with the predominant participation of COO^−^ groups of G residues. With an increase in the number of ions, M residues with the participation of OH groups are much more involved. It is possible that the number of Sr^2+^ ions, used in this work, was not sufficient to bind all the available alginate molecules. A larger number of Sr^2+^ binding sites with the alginate molecule, compared to Ca^2+^, was confirmed by the authors of [10]. The higher mechanical strength of Sr-alginate fibers, as well as FTIR data and circular dichroism spectroscopy results, contributed to the assumption about the formation of stronger Sr–O bonds [10]. In our work, a strong Sr–O bond was confirmed by a decrease in the resolution of the spectrum and the lower intensity of the signals (Figure 5B,C). 

**Figure 6:**
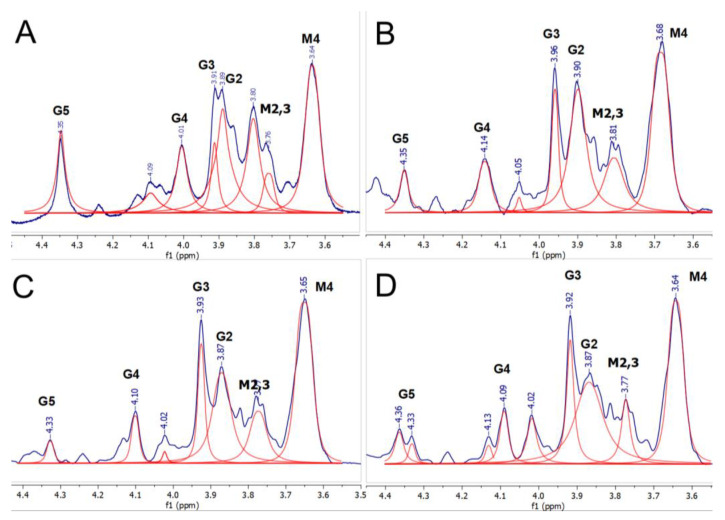
^1^H NMR spectra of ALG-Ca, cross-linked by different quantities (μmol) of Ca^2+^: (**A**) 0.3 μmol, (**B**) 2.5 μmol; (**C**) 5.5 μmol; (**D**) 25 μmol. Fitting is represented by red lines, fitting baseline is shown at the bottom.

In the spectra of ALG-Ca, with the increase in the ion concentration to 2.5 µmol, the intensity of G4 and G5 signals decreased (Figure 6). These experimental data account for a preferable formation of the “egg-box” structure. With a further increase in the quantity of Ca^2+^ ions up to 25 µmol, the intensity of signals did not change. Based on this, it can be assumed that all the available COO^−^ sites were occupied. With further addition of Ca, a decrease in the intensity and resolution of G2 and M2,3 signals, which correspond to protons adjacent to the OH group [28], was observed. The most energetically preferable for Ca^2+^ is COO^−^ sites, but when they are fully occupied, and the number of ions increases, Ca^2+^ ions begin to coordinate with OH groups. However, this position is not energetically preferred for Ca.

It can be concluded that despite the close energies of the ionic bonds of Ca–O and Sr–O in alginate, the coordination of these ions occurs in different ways. The most energetically favorable sites for Ca are GG residues with the formation of the “egg-box” structure, while only COO^−^ groups are involved in the Ca binding. On the other hand, Sr coordinates with both the COO^−^ groups and OH groups, and both G and M residues are involved. In this case, the cross-linking with Sr has a tighter spatial arrangement due to the presence of a larger number of binding sites.

### 3.2. EPR Results

EPR spectroscopic studies of the alginate solids containing paramagnetic copper (Cu) and manganese (Mn) ions were carried out. Cu–O and Mn–O are both characterized by the coordination bonds with alginate at similar binding energies [24].

**Figure 7:**
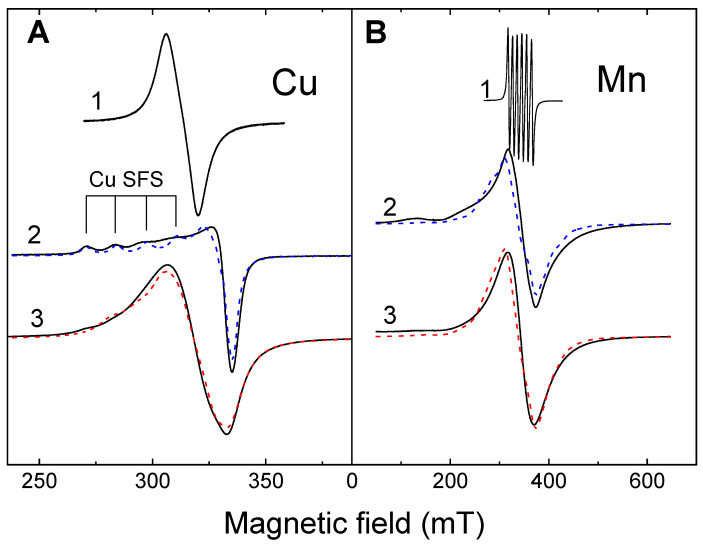
EPR spectra of Cu^2+^ (**A**) and Mn^2+^ (**B**) ions. 1—the EPR spectra of Cu(NO_3_)_2_ or Mn(CH_3_COO)_2_ solutions with a concentration of 102.5 mmol/L. 2—the EPR spectra of Cu^2+^ and Mn^2+^ ions in the alginate when the number of these ions is insufficient for complete cross-linking. 3—the EPR spectra of Cu^2+^ and Mn^2+^ ions in the alginate at an excess amount of these ions in the solution. The dashed lines show the modeling spectra.

**Figure 8:**
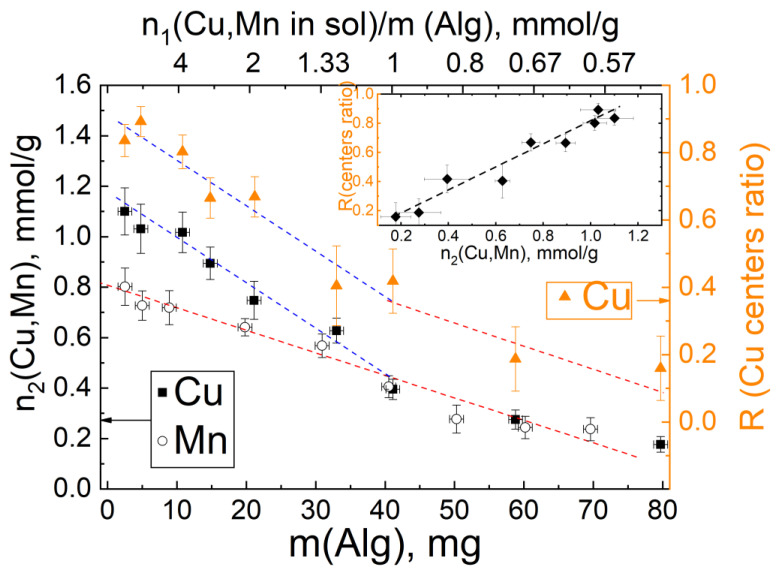
The measured value of concentration dependence (left axes) of Cu^2+^ (black filled square) and Mn^2+^ (black open circle) ions on the mass of ALG in 400 μL solutions of Cu(NO_3_)_2_ or Mn(CH_3_COO)_2_ with a concentration of 102.5 mmol/L. The ratio between the number of ions in the solution and the mass of the ALG sample (*n*_1_) is shown on top axis plots as recalculated bottom axes. The ratio *R* between the number of Cu pair centers and isolate Cu centers number (right axes) is shown as orange-filled triangles.

In the EPR spectra of Cu(NO_3_)_2_ or Mn(CH_3_COO)_2_ solutions, lines narrowed by the motion of the paramagnetic complex were observed (Figure 7, spectrum 1). An important parameter is the ratio between the cations number in the solution and the mass of ALG placed in the 400 μL solution; let us denote this parameter as *n*_1_ (Figure 8). After carrying out the cross-linking reaction in the dried samples, wider anisotropic EPR spectra were observed (Figure 7, spectra 2,3). In a standard routine described in [27] regarding the number of paramagnetic ions determination, the measured spectra were integrated twice for calculation of the spectrum area *I*_2_. The spectrum area *I*_2_ was divided into the Cu(DETC)_2_ spectrum area *I*_1_ and multiplied by the number of the Cu^2+^ ions in Cu(DETC)_2_ (*N*_1_ = 7.2 × 10^−9^ mol) for calculation of paramagnetic centers *N*_2_ according to the Equation (1),

(1) $$N_{2}=N_{1}\frac{I_{2}g_{1}S_{1}\left(S_{1}+1\right)}{I_{1}g_{2}S_{2}\left(S_{2}+1\right)}$$

where *g*_1,2_–*g*-factor, *S*_1,2_ paramagnetic ion spin (*S_Cu_* = 1/2, *S_Mn_* = 1/2). Then, *N*_2_ was normalized by sample mass in the *MW* cavity for paramagnetic centers concentration definition. The measured value of paramagnetic ion concentration was calculated as *n*_2_ (Figure 8, left axis). The dependence of the measured value of paramagnetic ions detection *n*_2_ on the mass of the ALG sample in solution *m*_1_ is shown in Figure 8. As can be seen from Figure 6, the selected range of *n*_1_ appears to be from 16 (left side of Figure 8) to 0.5 mmol/g (right side of the figure). Some of the unreacted Cu^2+^ or Mn^2+^ EPR ions were retained in the solution, so one can observe in Figure 8 a discrepancy between the cations’ quantity in the solution *n*_1_ and the cations’ quantity in the sample *n*_2_.

With a large excess of Mn^2+^ ions in the solution, the concentration *n*_2_ reaches 0.8 mmol/g. With a decrease in the number of Mn^2+^ ions in the solution, the concentration of ions in ALG–Mn also decreases (Figure 8, red dashed line), however, even at the smallest concentration, some of the Mn ions remain in the solution. It can be observed from Figure 7 that the EPR spectra of Mn^2+^ practically do not change, only the uniform width of the EPR line changes, in accordance with the change in the concentration *n*_2_. The shape of the spectrum is characteristic of the EPR spectrum of the Mn presence in solids [29]. The absence of the influence of a high concentration of paramagnetic impurity on the EPR spectrum indicates a weak interaction between Mn^2+^ ions; the ions are isolated from each other by alginate polymer molecules and do not form pairs. It can be assumed that the Mn^2+^ ions occupy an alginate structure with the largest distance between the neighboring ions, according to the scheme described for barium in [3] or according to the “pocket-like” scheme proposed in [21].

Despite similar energies of Mn–O and Cu–O bonds [24], at high *n*_1_ the cations form different structures in alginate.

In contrast to Mn^2+^ ions, the shape of the EPR spectra of Cu^2+^ ions in ALG depends on the number of Cu^2+^ cations in solution per 1 g of ALG, *n*_1_. For *n*_1_ = 0.5 mmol/g, a powder-like EPR spectrum was registered with a clear hyperfine structure due to the nuclear magnetic moment of Cu. The spectrum simulation with parameters of *g_xx_* = 2.067; *g_yy_* = 2.092; *g_zz_* = 2.387; *A_xx_,A_yy_* < 80 MHz; *A_zz_* = 368 MHz; and *g_zz_*/*A_zz_* = 194 is shown in Figure 7A as a blue dashed line. Such parameters correspond to single Cu^2+^ ions with octahedral coordination in the plane of oxygen ions [30]. As the relative number of Cu^2+^ cations *n*_1_ increased to 16 mmol/g, the EPR spectrum became more symmetrical, without a pronounced hyperfine structure. In [30,31,32], it was shown that such an EPR spectrum is characteristic of the appearance of Cu pairs. Electron magnetic moment of Cu^2+^ ions interacted by dipolar interaction (Equation (2)):

(2) $$eeD=\frac{\mu_{0}}{4\pi }\frac{\mu_{1}\mu_{2}}{r^{3}}$$

and triplet state occurred. The exchange coupling for Cu alginate complexes is usually blocked by oxygen ions of alginate molecules, so, it is not taken into account. Based on this assumption, the EPR spectrum of the Cu pair spectrum was simulated, it is shown as the red dashed line in Figure 7A. For comparison, the blue dashed line of a single Cu ion spectrum is presented. The dipole interaction energy is 150 ± 50 MHz and, in terms of the distance between Cu^2+^ ions in a pair, is about 7 Å. In the case of intermediate *n*_1_, the EPR spectrum contained both lines of paired and single Cu centers. By determining the integral intensities of each of the components, the relative number of paired centers depending on *n*_1_ was obtained, which we designated as *R* and shown in Figure 8 as orange triangles. As can be seen, the dependence of *R* on *n*_1_ repeats the dependence on *n*_2_. In the case of a high concentration of Cu^2+^ ion captured by the alginate polymer, paired centers can also form, however, due to the randomness of the process, their relative amount will be proportional to the square of the ion concentration. In our case (inset in Figure 8), a linear dependence was observed, which indicates that the formation of paired Cu centers is more energetically preferable to the formation of single centers, and with a sufficient number of Cu cations, this process is predominant.

Octahedral coordination is possible with the participation of not only carboxyl groups, but also OH and -O- groups of alginate [25]. In the octahedral coordination of Cu to the oxygens of alginate, taking into account the formation of paired centers, bridging oxygen atoms are formed [30]. Tighter spatial arrangement in cross-linking at higher concentrations provides higher viscosity [33]. 

It is of interest for future studies to further investigate the cross-linking of alginate with Cu^2+^ ions, since there have been no attempts in the available literature to perform DFT calculations involving such paired Cu centers, which, according to our obtained EPR data, are formed upon cross-linking with Cu ions.

## 4. Conclusions

The ^1^H NMR spectra obtained for alginate cross-linked with diamagnetic alkaline earth metal ions (Ca, Sr) and the EPR spectra for alginate cross-linked with paramagnetic transition metal ions (Cu, Mn) allowed us to draw the following conclusions. 

Ca and Sr form weak ionic bonds with the oxygen atoms of alginate, while Cu and Mn form strong coordination bonds. Based on this, similar behavior of Ca and Sr, and Cu and Mn is expected upon alginate cross-linking. However, the present study showed that this is not the case. The similarity of the proposed mechanisms was found, instead, for such pairs as Ca and Mn, and Sr and Cu ions.

As shown by the ^1^H NMR spectroscopy study, Ca occupies certain positions in the alginate structure, and coordination to other sites does not occur even if the number of ions is increased. The EPR spectra of Mn also showed no effect of concentration on the binding of Mn to alginate.

Sr and Cu occupy a larger number of possible positions, and this number increases with the increase in the ion concentration. In this case, Sr is coordinated to OH groups, while Cu forms pair centers, which indicates a close spatial arrangement of neighboring ions.

Thus, to obtain materials based on ionic cross-linked alginate, it is worth focusing mainly on the characteristics of specific ions. The data obtained in this work can be useful for the development of alginate materials with desired properties.

## Figures and Tables

**Table 1:** The cross-linking ion quantities used in the ^1^H NMR experiment.

Cation	Quantity, μmol
Ca^2+^	0.3; 1; 2.5; 5.5; 25
Sr^2+^	0.3; 1; 2.5
Zn^2+^	0.1; 0.3; 1
Co^2+^	0.1; 0.3; 1

## Data Availability

The experimental data on the results reported in this manuscript are available upon an official request to the corresponding authors.
